# 527 Bromelain Debridement’s Effect on the Surgeon’s Burn Wound Depth Assessment

**DOI:** 10.1093/jbcr/iraf019.156

**Published:** 2025-04-01

**Authors:** Alba Paulsen, Lucy Wibbenmeyer, Alexander Kurjatko, Robert Bertellotti, Shady Al Hayek, Siri Pothula, Colette Galet

**Affiliations:** University of Iowa; University of Iowa; University of Iowa; University of Iowa; University of Iowa; University of Illinois at Chicago; University of Iowa

## Abstract

**Introduction:**

Most surgeons rely on visual inspection to determine the healing capacity of burn wounds. For indeterminate wounds, this can create delays in care on one hand or unnecessary surgery on the other. We report herein the effect of bromelain debridement (BD) on the assessment of indeterminate wounds.

**Methods:**

Three surgeons reviewed 92 images (46 pre- and 46 post-BD treatment) of burn wounds from 31 patients who were previously treated with BD as part of the NEXT study (Mediwound). The images were labeled pre or post BD treatment but were otherwise deidentified and placed in random order. Each surgeon was asked to estimate wound depth defined as the percent superficial partial (SPT), deep partial (DPT), and full thickness (FT), time to heal (< 1, 1, 2, or ≥3 weeks), and if grafting would be required. Intra class correlation (ICC) was used to assess inter-rater agreement for SPT, DPT and FT ratings as well as time to heal. P < 0.05 was considered significant.

**Results:**

Most patients were male (64.5%), white (68.6%), and young (mean 37 years of age). Most suffered flame burns (64.5%); the median burn size was 8.3%. Twenty-five patients had one wound and six had two or more wounds treated with BD. Grafting was performed on 20 wounds on a total of 11 patients. Wounds not grafted healed significantly faster than those grafted (2 [IQR 1-3.25] vs. 4 [IQR 2.25-5] weeks; p < 0.001) with a significantly lower Modified Vancouver Scar Scale (MVSS) score (3 [range 0 to 10] vs. 5 [range 1-14]; p = 0.024). Interrater agreement increased significantly post-BD treatment for SPT and DPT assessments compared to pre-BD treatment (ICC 0.864 and 0.876 respectively, p < 0.001 compared to -0.052 and -0.689 respectively, p = 0.567 and 0.973). BD treatment also improved the interrater agreement when estimating time to heal pre- and post-BD treatment (ICC = 0.539 pre-BD vs 0.652 post). BD treatment did not improve the agreement on the need for skin grafting which remained slight pre- and post-BD treatment (ICC = 0.213 and 0.227, p = 0.012 and 0.008).

**Conclusions:**

BD treatment significantly increased the agreement between surgeons on estimating partial thickness wounds and time to heal. However, it did not clarify need for a skin graft. As the latter is largely subjective and based on several factors, we postulate a larger homogenous rater pool may be needed to assess whether BD treatment improves surgeons’ ability to estimate the need for skin grafting.

**Applicability of Research to Practice:**

BD may increase the ability of surgeons to estimate wound depth and time to heal. More research is needed to determine if it effects surgical decision making.

**Funding for the Study:**

N/A

